# Central Mirror Foot: Treatment and Review of the Literature

**DOI:** 10.7759/cureus.8448

**Published:** 2020-06-04

**Authors:** Yvonne-Mary Papamerkouriou, Georgia Antoniou, Panayotis Krallis, John Anastasopoulos

**Affiliations:** 1 Orthopaedics, Panagiotis & Aglaia Kyriakou Children's Hospital, Athens, GRC; 2 Orthopaedics, Evangelismos General Hospital, Athens, GRC; 3 Orthopaedics, Agia Sofia Children's Hospital, Athens, GRC

**Keywords:** mirror foor, central ray, polydactyly

## Abstract

Mirror foot is a rare abnormality which presents as a preaxial, postaxial, or central polydactyly of the foot. The latter is encountered infrequently. We describe the case of a central mirror foot. Our patient had eight digits of a central ray pattern type with fully developed metatarsal, proximal, middle, and distal phalanges, as well as a medial toe syndactyly. He had no tarsal bone duplications. He was treated by central ray resection via double V-shaped incisions on the dorsal and plantar aspects of the foot, while preserving the medial and lateral rays. The results were satisfactory. We describe the technique and attempt a review of the literature.

## Introduction

Mirror foot is a rare abnormality associated with polydactyly. Polydactyly is postaxial when the extra ray is on the lateral side of the foot (almost 79% of the time) and preaxial (15%) when the polydactyly is on the medial (tibial) side of the limb [1,2]. Central duplication is quite rare, comprising 6% of the cases [2]. The mirror-image foot, with the full duplication of the foot rays, is uncommon. It is often reported along with other leg anomalies [3-5]. Treatment consists of excision of the extra rays to allow fitting of shoes [6]. This is often done via racket-type incisions of the border extra digits. The most normal-looking digits are usually preserved. In the central type of the mirror foot, the resection of the middle rays will produce a functional and cosmetic foot [7]. We present a case of central mirror foot treated surgically by dorsal and plantar V-shaped incisions. This surgical technique has only been reported once more in the literature.

## Case presentation

**Figure 1:**
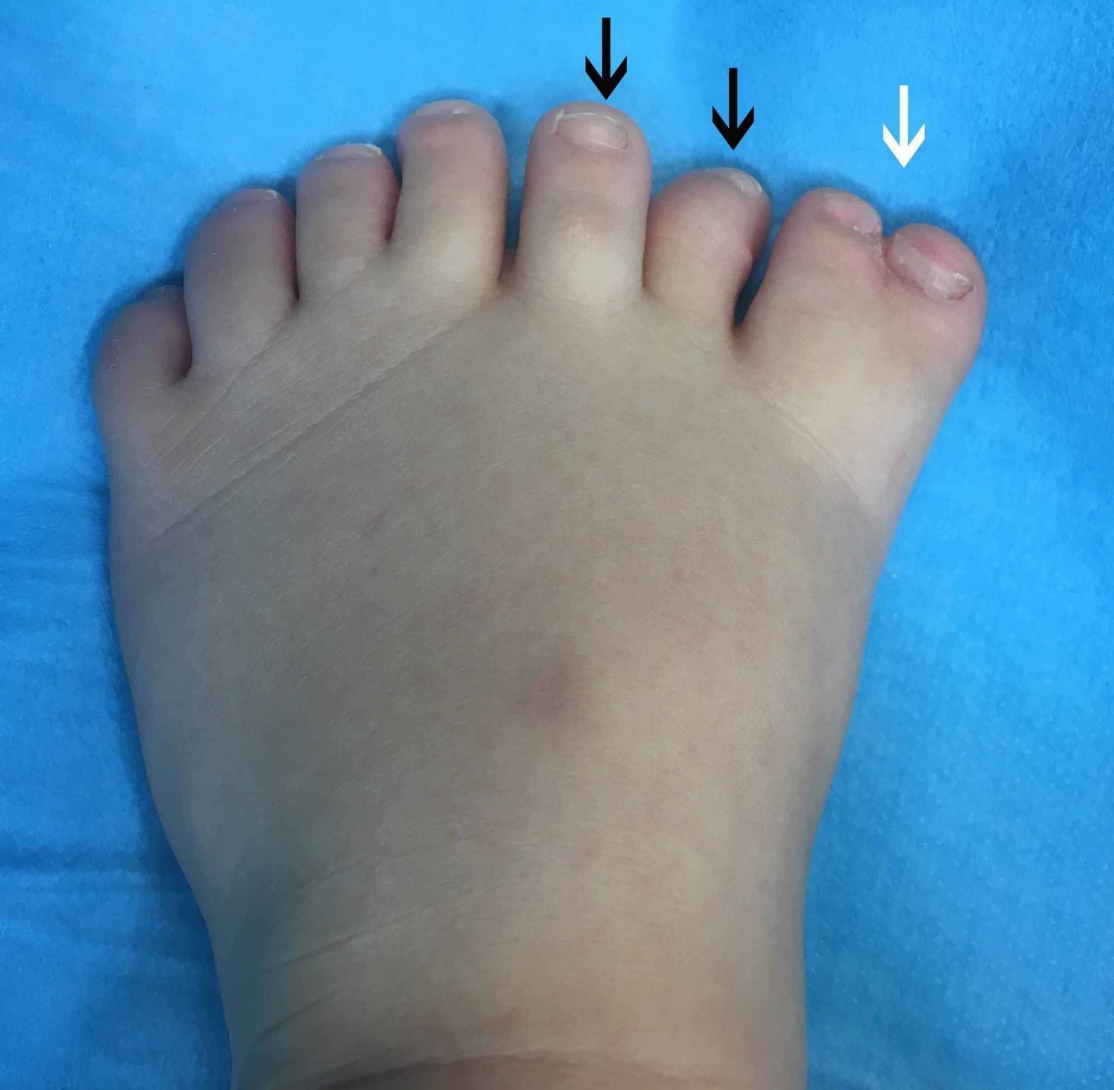
Left central ray mirror foot, dorsal side Left central ray mirror foot with medial toe syndactyly (white arrow) and two more extra digits (black arrows).

**Figure 2:**
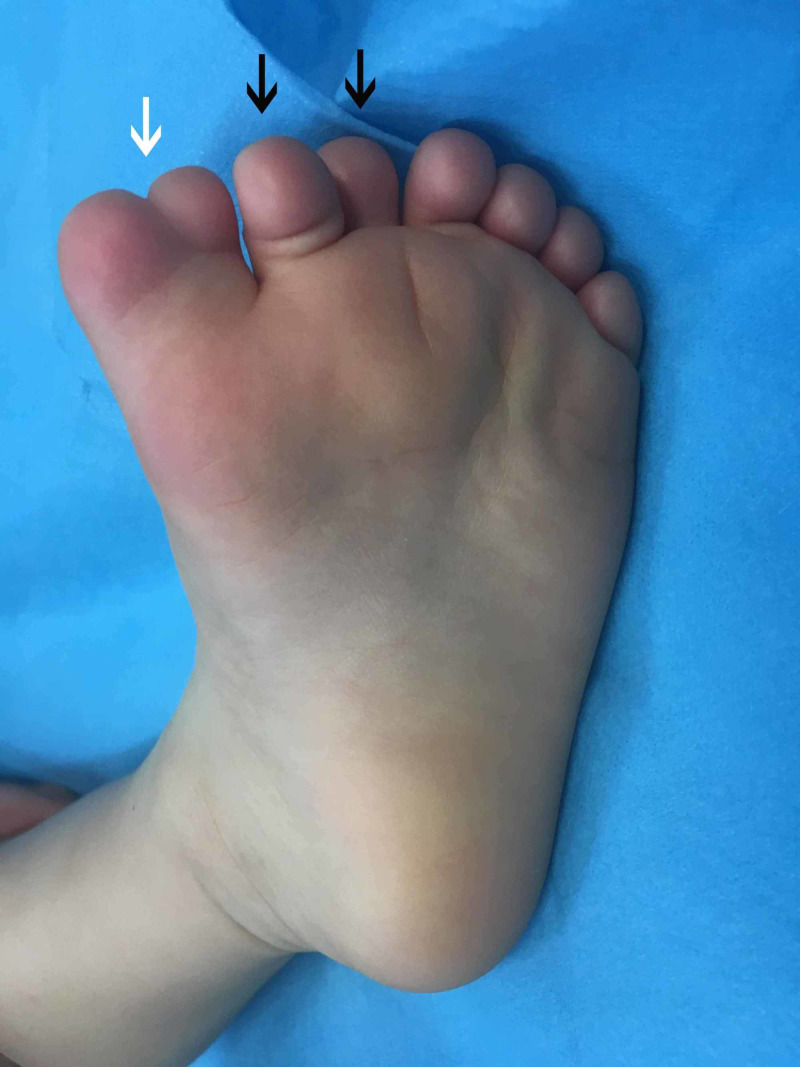
Left central ray mirror foot, plantar side Left central ray mirror foot with medial toe syndactyly (white arrow), and two more extra digits (black arrows).

**Figure 3:**
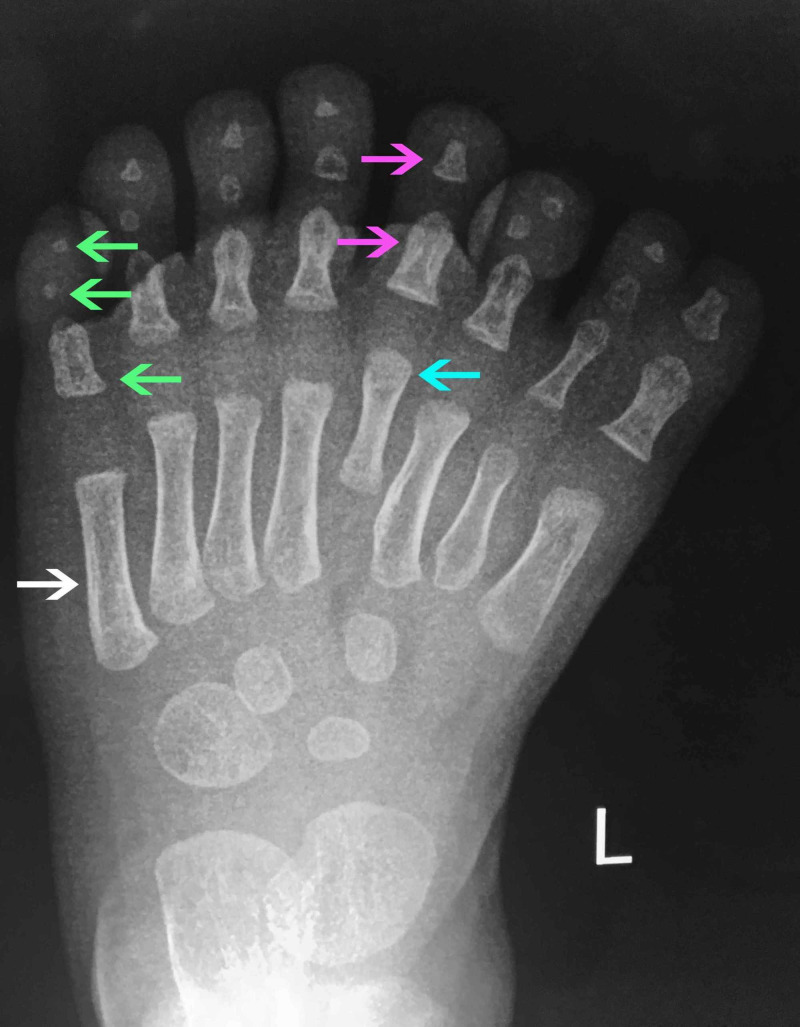
X-ray (XR) left central ray mirror foot XR left central ray mirror foot with eight digits portraying fully developed metatarsal (white arrow), proximal, middle, and distal phalanges (green arrows), except for the fourth toe from the medial side which appeared to be missing a middle phalanx (pink arrows) and had a dysplastic metatarsal (blue arrow), in comparison to the rest (white arrow). This digit was similar to a great toe.

A two-year-old boy with a left mirror foot was referred to our orthopaedic department. He was born full-term by natural delivery and was the firstborn of the couple. There was no family history of similar skeletal abnormalities and there was no history of drug or radiation exposure during gestation. The clinical and radiological assessment did not reveal any other skeletal abnormalities. The child had eight toes of a central ray pattern with a medial toe syndactyly (Figures 1, 2) with fully developed metatarsal, proximal, middle, and distal phalanges, except for the fourth toe from the medial side which appeared to be missing a middle phalanx and had a dysplastic metatarsal. This digit was similar to a great toe (Figure 3).

**Figure 4:**
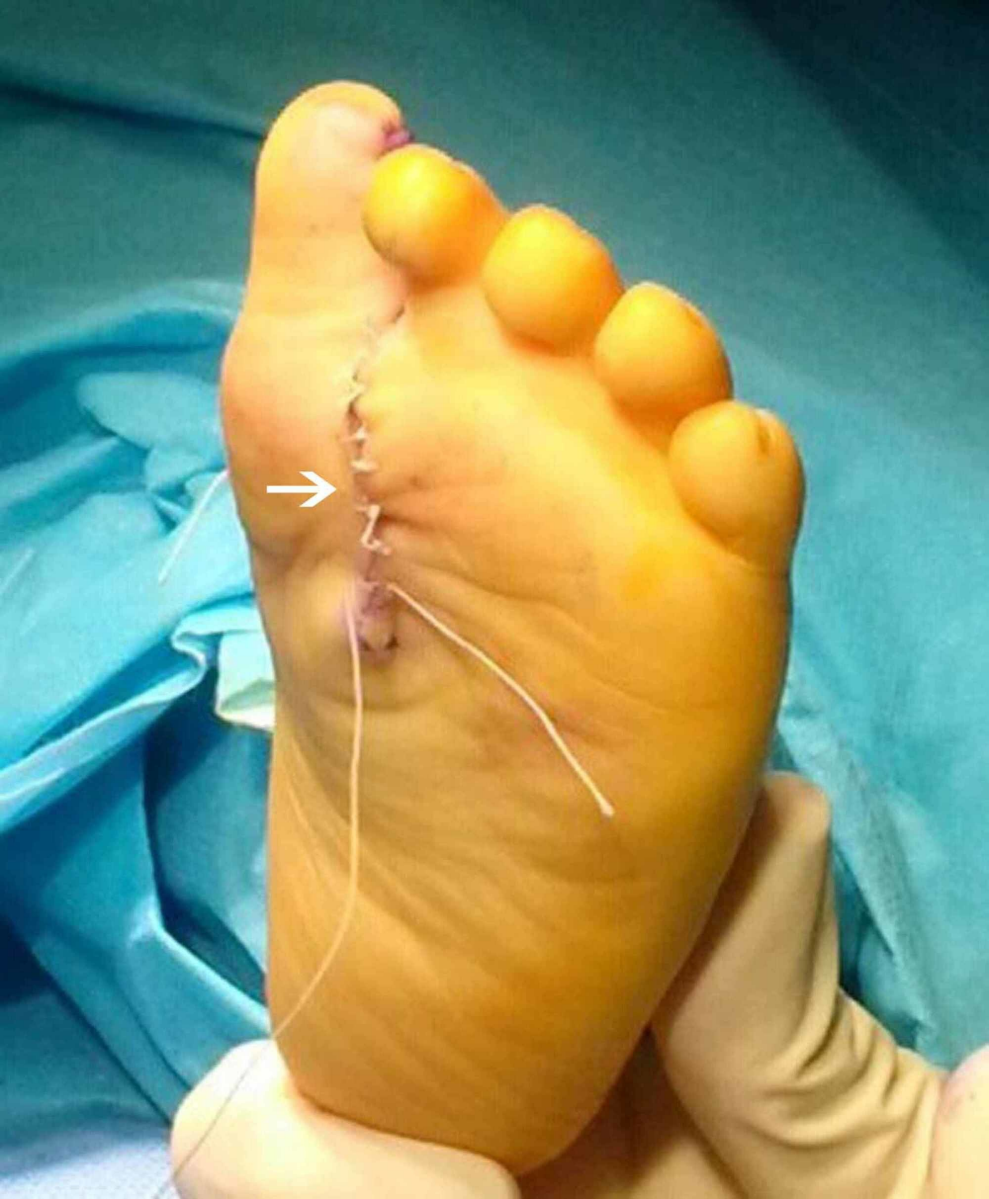
Postoperative photo, plantar view Postoperative photo indicating plantar incision (white arrow).

**Figure 5:**
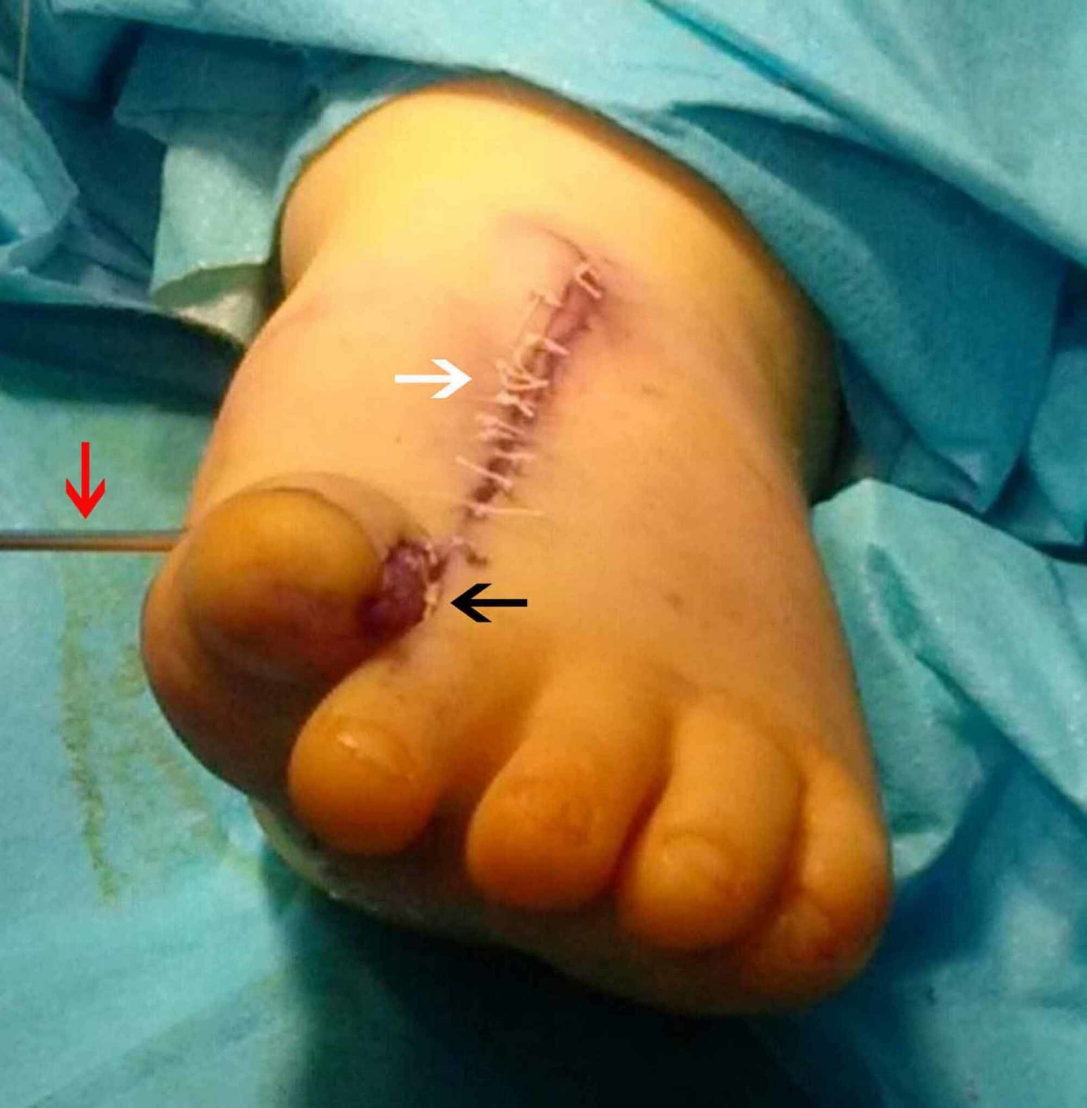
Postoperative photo dorsal view Postoperative photo dorsal view where dorsal incision is evident (white arrow), medial syndactyly has been separated and plantar flap has been used for coverage (black arrow). K-wire was inserted for stabilisation (red arrow).

Surgery was performed under general anaesthesia, and a tourniquet was applied. There was a medial toe syndactyly and the fourth toe from the medial side was abnormal; therefore, we decided to remove the second, third, and fourth toe from the medial side. Simultaneous dorsal and plantar V-shaped incisions were made in the centre of the foot, and the extra skin and three central rays were removed. The extra tendons and digital nerves were resected, and the nerve stumps were cauterised by diathermy in order to prevent a neuroma. The middle cuneiform was not removed to avoid tarsal instability. There were no tarsal bone duplications, and therefore no tarsal bones were removed. The removal of the second toe from the medial side meant an inevitable disruption of the Lisfranc ligament. Therefore, the remaining adjacent metatarsals were approximated using a percutaneous K-wire, inserted through the first metatarsal. Furthermore for the restoration of stability, the intermetatarsal ligaments were sutured. A plantar skin flap from the excised second medial toe was used for covering the lateral surface of the first medial big toe (Hallux). The skin was sutured with an interrupted rapid suture (Figures 4, 5). A below-knee cast was applied which was removed at four weeks. At that point, the K-wire was also removed.

**Figure 6:**
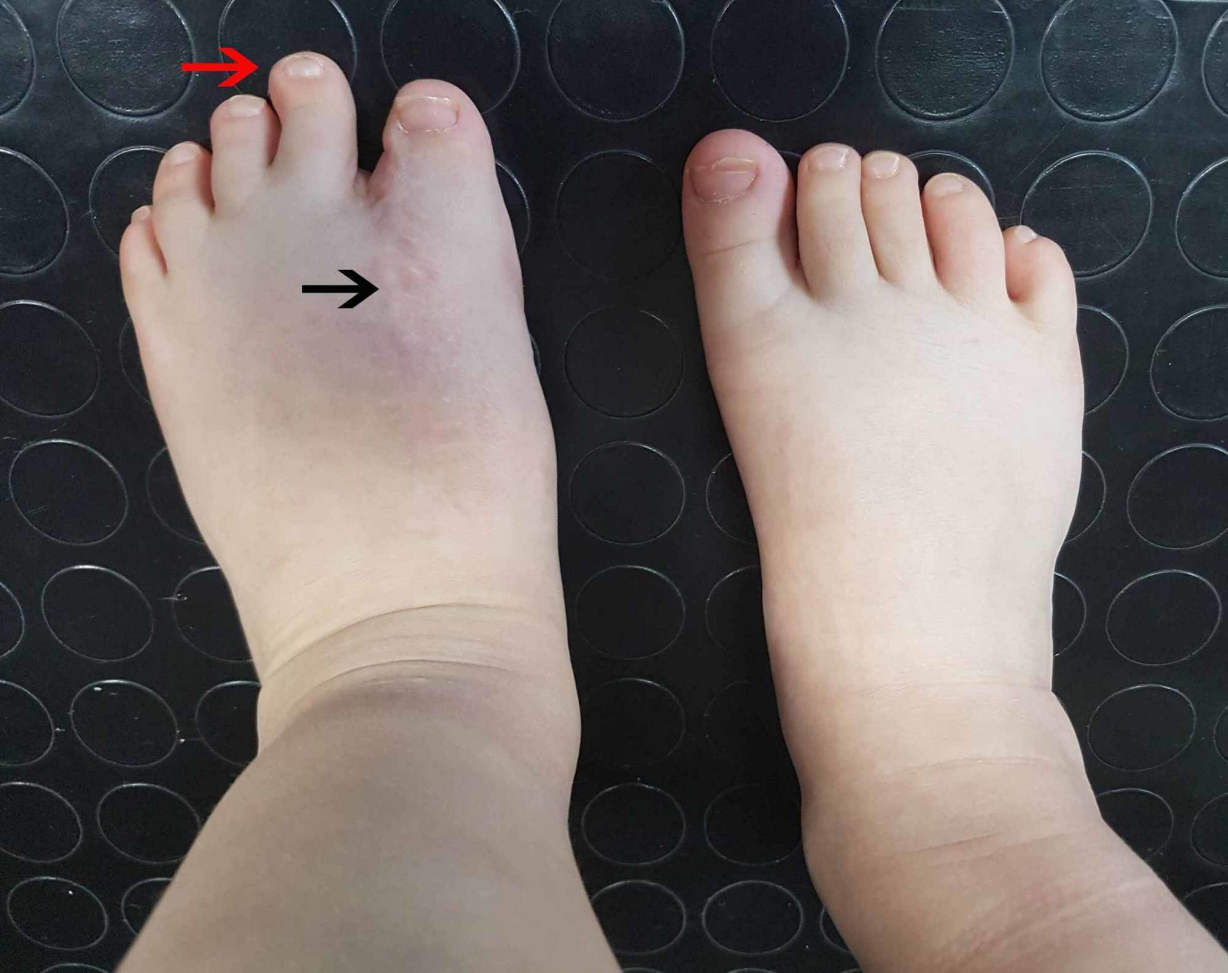
Two-year postoperative photo Two-year postoperative photo where the surgical scar is hardly evident (black arrow) and the left foot has a comparable width to the normal right foot. The second toe, however, is considerably larger than normal side (red arrow).

**Figure 7:**
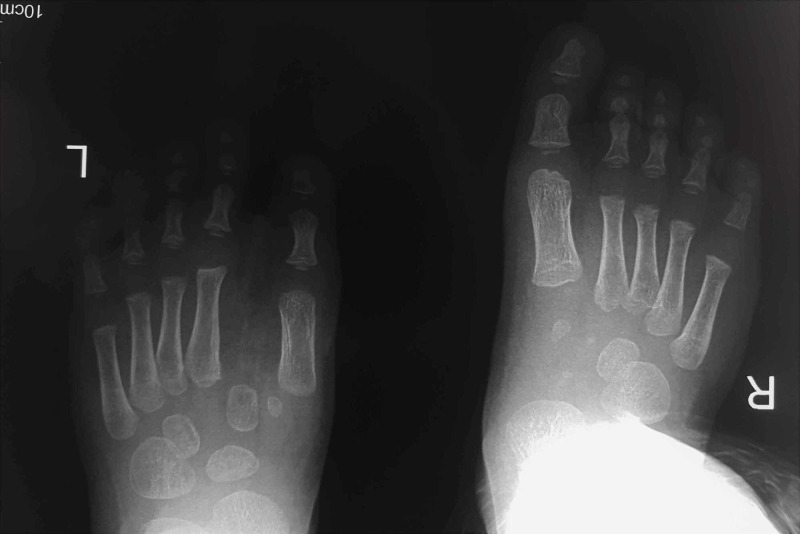
Two-year follow-up X-ray (XR) Two-year follow-up XR where a mild widening of the middle foot is apparent.

The patient was followed up two years postoperatively and examined both clinically and radiologically (Figures 6, 7). There was a mild widening of the left foot; however, there was no clinical instability. We would consider the possibility of removing the middle cuneiform at a later stage, were complications to arise. At the time of evaluation, the result was overall satisfactory. The patient had a plantigrade foot and was able to walk with normal shoes, without a limp. Additionally, he could actively dorsiflex his foot and extend his great toe. Also, he could walk on tiptoes.

## Discussion

The definition of mirror foot is diverse, due to the variability in patterns. Also, different terminologies are used to describe this specific type of foot duplication. The most common terms are diplopodia and mirror (-image) polydactyly [6]. In a paper published in 2016 presenting a review of the literature, when these terms were included, 78 patients, with 118 mirror feet, were identified in total. In 97 feet, the tibial development was evaluated and in 74.2% of the cases, it was abnormal. In 55 feet, the tarsal region was evaluated, and in 94.5% of these cases, it showed abnormalities [6]. Of the 78 patients, only nine had a follow-up period of more than five years and of the 118 feet, in only four feet were complaints recorded on the long term due to osseous abnormalities [8-10].

Lateral or postaxial polydactyly is the most common form of foot polydactyly, and surgical correction is performed with the most lateral being disarticulated in most cases by a racquet incision [11]. Management of medial polydactyly included preservation of the digit with the best axial alignment, resection of the projecting and symptomatic toe, correction of the alignment of the residual great toe, rebalancing of the soft tissues, reduction of the medial metatarsal prominences, and provision of adequate, but not excessive, soft tissue coverage (Mod McElvenny procedure, Farmer’s type first web reconstruction, medial Z-plasty of skin) [2].

In central polydactyly, a surgical technique describing plantar and dorsal advancement flaps has been described by Allen [12]. When the second ray is duplicated, laterally based dorsal and plantar flaps are developed, beginning at the medial margin of the forefoot and extending distally along the great toe to the level of the first web space. When the fourth ray is duplicated, medially based dorsal and plantar flaps are developed, beginning at the lateral margin of the foot and extending distally along the small toe to the level of the first web space. A retrospective case series of 22 patients with 27 feet with central polydactyly, treated by the above-mentioned technique, reported excellent results [13].

Another surgical technique utilised in central polydactyly is wedge excision through a dorsal racquet incision. All of the studies describing this technique mention persistent widening of the forefoot after surgical management of the central polydactyly [14-16].

We used a different technique consisting of a combination of dorsal and plantar V-shaped incisions allowing the removal of three rays (second, third, and fourth from the medial side) as well as the extra skin. This has only been described once in the literature, in a report of two cases [7]. The result was a functional and cosmetic foot enabling shoe wear.

## Conclusions

Central ray mirror foot is the most uncommon form of this rare deformity. Few cases have been reported, and there is little consensus concerning the optimal technique for surgical management. We had satisfying clinical and radiological results after the treatment of a two-year-old with central ray mirror foot treated with a combination of V-shaped dorsal and plantar incisions.
